# Insights from expert elicitation: prioritising pathogens for genome sequencing for Australian public health response

**DOI:** 10.1016/j.lanwpc.2025.101670

**Published:** 2025-08-25

**Authors:** Tehzeeb Zulfiqar, Angeline Ferdinand, Danielle Cribb, Patiyan Andersson, Alireza Zahedi, Kathryn Glass, Son Nghiem, Susan Trevenar, Nhung Mai, Benjamin P. Howden, Martyn D. Kirk, AusPathoGen Program Partners

**Affiliations:** a Department of Applied Epidemiology, National Centre of Epidemiology and Population Health, The Australian National University, Canberra, Australia; b Microbiological Diagnostic Unit Public Health Laboratory, Department of Microbiology and Immunology, The University of Melbourne at The Peter Doherty Institute for Infection and Immunity, Melbourne, VIC, Australia; c Microbiological Diagnostic Unit Public Health Laboratory, The University of Melbourne at the Peter Doherty Institute for Infection and Immunity, Melbourne, VIC, Australia; d Department of Microbiology and Immunology, The University of Melbourne at The Peter Doherty Institute for Infection and Immunity, Melbourne, VIC, Australia; e Centre for Pathogen Genomics, The University of Melbourne, Melbourne, VIC, Australia; f Public Health Microbiology, Public and Environmental Health Reference Laboratory, Pathology Queensland, Queensland Public Health and Scientific Services, Queensland Health, Brisbane, Australia; g Centre of Epidemiology, Policy and Practice, National Centre of Epidemiology and Population Health, The Australian National University, Canberra, Australia; h Department of Health Economics Wellbeing and Society, National Centre of Epidemiology and Population Health, The Australian National University, Canberra, Australia; i Centre for Health Services Research, The University of Queensland, Brisbane, Australia; j Department of Infectious Diseases, Austin Health, Heidelberg, Melbourne, VIC, Australia

**Keywords:** Expert elicitation, Delphi method, Pathogens, Prioritisation, Genome-surveillance, Public health response, Infectious disease

## Abstract

**Background:**

Pathogen genomics has transformed infectious disease response, yet prioritisation frameworks for genome sequencing remain underdeveloped. This study establishes evidence-based criteria for prioritising pathogens for genome sequencing to maximise public health impact in Australia.

**Methods:**

We conducted a modified Delphi study with experts in public health, infection prevention, and pathogen genomics to examine both prioritisation mechanisms, selection criteria and specific pathogen rankings across surveillance contexts. Initially, 38 experts evaluated 89 statements on a 5-point Likert scale with accompanying feedback. In round two, participants reassessed 48 statements, including 28 that were revised based on first-round input. Quantitative data was analysed using STATA-18 and qualitative data using Atlas.ti.

**Findings:**

Consensus was achieved on 53 statements across both rounds across three domains: decision-making processes, prioritisation criteria, and high-priority surveillance scenarios to prioritise specific pathogens for genome sequencing. Experts agreed that a national priority pathogen list for genome sequencing should be developed collaboratively with public health laboratories and complemented by state level lists, with biennial reviews and flexibility for situation-based adjustments. Consensus was achieved on prioritising pathogens associated with antimicrobial resistance, novel and emerging potential, virulence, institutional transmission risk, and disproportionate impact on Aboriginal and Torres Strait Islander communities. For routine surveillance, *Mycobacterium tuberculosis* received highest consensus for sequencing, followed by multidrug-resistant *Staphylococcus aureus*. Eleven pathogens were prioritised for sequencing in outbreak investigation, including Carbapenemase producing *Enterobacterales*, pathogenic *Escherichia coli* subtypes, and *Salmonella* species. Three pathogens, *Shigella*, *Neisseria gonorrhoeae*, and invasive Group A *Streptococcus*, were prioritised for periodic surveillance sequencing. Our qualitative analysis showed experts emphasised public health significance and actionability while advocating for balanced national-local governance and cross jurisdictional collaboration to maximise resources.

**Interpretation:**

This study establishes foundational evidence for developing a comprehensive framework for prioritising pathogens for genomic sequencing in Australian public health surveillance and response.

**Funding:**

Australian National Health and Medical Research Council, Medical Research Futures Fund (FSPGN00049).

**Research in context**

**Evidence before this study**

No established criteria exist for prioritising pathogens for genomic surveillance in public health response. Most initiatives respond to specific threats rather than using systematic frameworks.

**Added value of this study**

We provide consensus-based criteria for prioritising pathogens for genomic surveillance in Australian public health, considering pathogen characteristics, epidemiological factors, and implementation considerations. We identified priority pathogens for different surveillance activities and governance requirements balancing national coordination with jurisdictional autonomy.

**Implications of all the available evidence**

Our findings provide a foundation for evidence-based prioritisation frameworks that can guide resource allocation and be adapted for different settings. Regular reviews would ensure surveillance remains responsive to emerging threats and evolving technologies.

## Introduction

Pathogen genomics represents a transformative leap in infectious disease surveillance, enabling unprecedented precision in detecting both novel and re-emerging infectious diseases, mapping transmission networks, and monitoring antimicrobial resistance (AMR).^1^ It provides detailed genetic information beyond traditional diagnostics, reveals relationships between cases by tracking variants, and offers crucial insights into resistance mechanisms and transmission patterns.^1^^,^^2^ When integrated with surveillance data, pathogen genomics allows more accurate outbreak source identification and transmission tracking across populations and geographical boundaries^3^; capabilities that have become essential for managing infectious diseases and containing the spread of drug-resistant pathogens.

Recognising the capabilities of pathogen genomics, the World Health Organization's (WHO) “Global Genomic Surveillance Strategy (2022–2032)” established a framework for strengthening global health security by expanding genomic surveillance to enable timely and effective public health actions at local, national and global levels.^4^ While this global strategy provides broad direction, individual countries need tailored implementation approaches that consider their specific contexts and resources.

As genomic technologies become more accessible, health systems worldwide face a critical challenge of determining which pathogens should be sequenced due to the limited resources of laboratories. From approximately 1415 species capable of infecting humans,^5^ surveillance systems target those posing significant population health threats. This selective approach is reflected in national surveillance programs, with the United States (US) monitoring 120 pathogens,^6^ Canada 56^7^ and Australia 65 pathogens.^8^ Given the high cost and specialised expertise required for genomic sequencing, prioritisation is essential to maximise public health impact and ensure efficient resource allocation.

Pathogens have been selected for surveillance using various criteria: public health impact including disease burden and mortality^9^, ^10^, ^11^; epidemiological factors such as incidence and outbreak potential,^9^^,^^10^^,^^12^ antimicrobial resistance,^11^^,^^13^ economic impact,^14^ and zoonotic potential.^15^ As genomic technology emerges, evidence-based criteria for pathogen prioritisation are needed to guide public health decision-making and targeted interventions. However, no consensus currently exists on standardised methods for pathogen prioritisation, underscoring the need for systematic consensus-building approaches.

Various methodologies have been used to prioritise pathogens and disease risks. These include quantitative scoring methods such as Bibliometrics (rank pathogens based on publication frequency and citation impact), the Delphi technique (a structured iterative process gathering expert consensus), multi-criteria decision analysis (MCDA) (combining multiple weighted criteria into a single prioritisation score), questionnaires (standardised surveys collecting data across predefined categories), and multi-dimensional matrices (frameworks evaluating pathogens against multiple variables simultaneously).^15^ Among these, MCDA and the Delphi technique are generally considered the most robust and widely used approaches for pathogen prioritisation by globally important health organisations such as the WHO, the European Centre for Disease Prevention and Control (ECDC), and the US Centers for Disease Control and Prevention.^12^, ^13^, ^14^^,^^16^ The strength of MCDA lies in quantitatively ranking alternatives against established criteria with explicit weightings.^14^ The Delphi technique proves valuable for questions beyond experimental and epidemiological methods, where empirical evidence is scarce and predictions, shared priorities, or concept definitions are needed.^14^^,^^17^ It facilitates consensus through iterative rounds of expert feedback, reducing bias while accommodating diverse expertise—qualities that make it particularly well-suited for complex public health questions^17^^,^^18^ and Australia's federated health system.

Australia's federated health system, where states and territories manage public health activities and response independently,^19^ presents unique challenges for genomic surveillance. Australia established initiatives such as the Communicable Diseases Genomics Network (CDGN) of public health laboratories (2015)^20^ and AusTrakka (2020) for secure genomic data sharing between states and territories and between Australia and New Zealand.^19^ In 2025, Australia revised the National Microbial Genomics Framework^21^ to address key governance issues related to pathogen genomics. In 2021, Australian public health reference laboratories and academic partners established AusPathoGen (AusPathoGen; 2021–25) funded under the Australian Governments Medical Research Future Fund. AusPathoGen aimed to support the integration of pathogen genomics into public health in an equitable and nationally consistent way.^20^ This study, conducted as part of AusPathoGen, provides evidence-based mechanisms and criteria for pathogen prioritisation that can be implemented consistently across all Australian jurisdictions.

This research aims to establish a consensus-based framework for prioritisation using a modified electronic Delphi methodology.^18^ The goal is to ensure genomic sequencing resources target pathogens with the greatest public health impact in Australia. We evaluated both general prioritisation criteria and specific pathogen rankings across different surveillance contexts to provide comprehensive guidance for implementation. We selected the Delphi approach due to limited empirical evidence on pathogen prioritisation for genomic surveillance and the need to systematically integrate diverse expert perspectives across Australia's federated health system. The method's strength in building consensus through structured feedback,^17^ made it ideal for developing this framework where quantitative data alone would be insufficient.

## Methods

### Establishing priority statements

We conducted a rapid review of published literature from January 2010 to November 2022 in PubMed, Scopus, and Web of Science that identified 14 publications on prioritisation approaches for genomic surveillance. Our search strategy used combinations of key terms including ‘pathogen genomics’, ‘whole genome sequencing’, ‘surveillance’, ‘prioritisation’, and ‘public health response’. We included peer-reviewed publications and published reports in English that described criteria, frameworks, or approaches for selecting pathogens for genomic surveillance. We excluded publications focused solely on technical aspects of sequencing without relevance to prioritisation decisions.

From these publications, we developed 32 broad statements about the role of pathogen genomics in public health responses. Our research team reviewed and refined these statements, creating a final list of 89 statements across three domains^1^: decision-making mechanisms and processes,^2^ criteria for prioritisation, and^3^ situations warranting highest-priority genomic surveillance.

We focused on 14 priority pathogens identified by the AusPathoGen program due to their high disease burden, frequency as a cause of outbreaks, difficulty in investigation and control, spread to high-risk and vulnerable populations, or resistance to almost all available antimicrobials.^20^ Based on research team discussion, we included both *E. coli* as a broad category and specific pathogenic *E. coli* subtypes (STEC, ETEC, EIEC, EAEC) as separate entries in Round 1, resulting in 15 pathogen entries for expert evaluation, recognising that these pathotypes have distinct epidemiological and virulence characteristics.^22^ Invasive Group A Streptococcus (iGAS) was added in Round 2 based on expert suggestions from Round 1 free-text responses. A list of these pathogens is provided in the Supplementary Material.

### Participant recruitment

We invited 83 Australian experts with experience in infectious disease surveillance, public health and pathogen genomics (identified from the AusPathoGen stakeholder's directory) to participate in the study. Participants represented diverse sectors including public health laboratories and public health units from all jurisdictions, CDGN, the Public Health Laboratory Network, the Communicable Disease Network Australia, animal health, environmental science, Department of Agriculture, Commonwealth Scientific and Industrial Research Organisation, the OzFoodNet (Australian foodborne diseases surveillance network), and academia.

### Delphi process

**Fig. 1:**
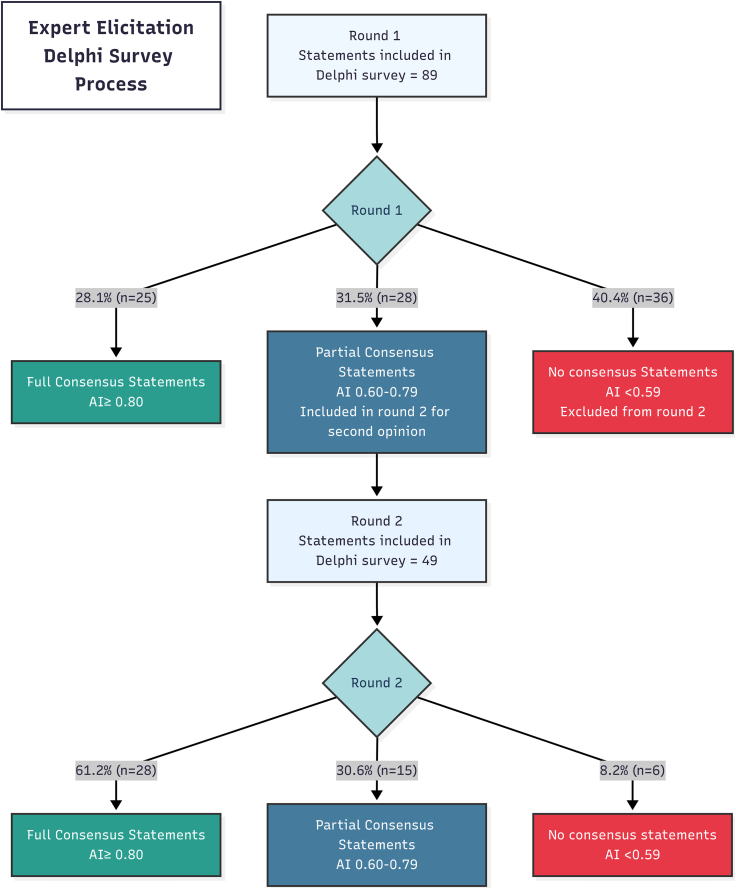
Two-round Delphi process with agreement index thresholds. Agreement Index (AI) is a measure of consensus where 1 represents perfect agreement. In this Delphi survey cutoff for Full consensus were set at AI > 0.80; Partial consensus: AI 0.60–0.79 and No consensus: AI < 0.59.

We developed the Delphi survey in REDCap (v14.7.3). We pilot-tested the survey with two experts to evaluate its language, terminology, flow, completion time, and clarity. To minimise survey fatigue, participants were able to complete the survey across multiple sessions. After incorporating their feedback, we conducted two rounds of the online survey from May 2023 to April 2024 (Fig. 1).

In round 1, experts rated 89 statements on 5-point Likert scales: for mechanisms and processes (1 = strongly agree to 5 = strongly disagree), general prioritisation criteria (1 = extremely important to 5 = not important), and practical application of prioritisation expertise to 15 specific pathogens across three contexts]—outbreak investigation, routine surveillance, and periodic surveillance (1 = highest to 5 = lowest priority). In Round 2, experts evaluated 16 pathogen entries following the addition of invasive Group A Streptococcus based on Round 1 expert suggestions. These components were part of a single integrated study: the first two question types established prioritisation criteria and processes, while the third applied expert judgement to rank predetermined pathogens across surveillance contexts. The consensus statements and pathogen rankings were complementary but independent components—the consensus statements established theoretical prioritisation frameworks while the pathogen rankings demonstrated practical application of expert judgement, rather than the consensus statements being used to directly rank the 14 pathogens.

Statements were organised by domain to maintain logical progression rather than randomised order, as our completion rates (38 experts in Round 1, 28 in Round 2) suggested survey fatigue was not a significant barrier.

We defined outbreak investigation as genomic sequencing to identify the source of ongoing outbreaks and prevent additional cases. Routine surveillance was defined as regular, ongoing systematic collection, analysis, and interpretation to monitor circulating pathogens and detect changes or trends over time. Periodic surveillance involved one-time analysis of representative samples at a specific point in time to determine what pathogens are currently circulating in a population.^23^ For all statements, experts could select “no opinion” and provide comments in free-text fields.

In round 2, experts received analysis reports comparing their ratings with group results and rated partial consensus statements plus 21 new statements derived from round 1 comments. Statements that achieved consensus in Round 1 were not presented again in Round 2. The Delphi study concluded after round 2.

### Data analysis and reporting

Our quantitative analysis followed “Conducting and Reporting of Delphi Studies” (CREDES) guidelines.^24^ We measured consensus using an Agreement Index (AI), defined as the proportion of experts who agreed or disagreed with each statement. We established three consensus levels: full (AI ≥0.80), partial (AI 0.60–0.79), and none (AI <0.59). This approach captured both strong agreement and strong disagreement; when ≥80% of experts disagreed with a statement, it was recorded as full consensus but reported with a negative direction. Statements with AI <0.59 indicated divided expert opinions rather than unified disagreement. We adopted this stringent threshold to ensure recommendations would have sufficient credibility to influence prioritisation frameworks, aligning with policy-relevant Delphi best practices.^17^ We excluded “no opinion” responses from analysis and identified new statements or revisions from round 1 free-text responses for round 2. Quantitative data was analysed using STATAMP.v18.

We also conducted thematic analysis of the free text responses to contextualise ratings in ATLAS.ti 8. After coding responses, we grouped similar concepts, identified themes, and organised relationships between themes to understand the context of experts’ ratings. For methodological rigor, we employed data triangulation by systematically comparing quantitative consensus results with qualitative themes, identifying areas of convergence, and complementarity to strengthen the validity of our findings.^25^

### Role of the funding source

Australian National Health and Medical Research Council, Medical Research Futures Fund (FSPG N00049), and Investigator Grant (GNT1196103) to BPH. The funding was provided to support activities of the AusPathogen project, including this study.

## Results

Thirty-eight experts participated in round 1 and 28 in round 2 (Table 1). Most experts in round 1 were epidemiologists followed by laboratory directors and physicians. All Australian jurisdictions except Western Australia were represented in round 1, while round 2 also lacked representation from Tasmania. A majority of participants held a PhD, and approximately two thirds had over 10 years' experience in infectious disease control.

**Table 1:** Expert participant profiles: rounds 1 and 2.

Participant characteristics	Number in round 1 (*n* = 38)	Number in round 2 (*n* = 28)
**Job titles at current place of employment** (***n***, %)		
Epidemiologists	12 (31.6)	9 (32.1)
Laboratory director	8 (21.1)	6 (21.4)
Physicians	5 (13.2)	2 (7.1)
Microbiologists	4 (10.5)	4 (14.3)
Senior scientists	4 (10.5)	4 (14.3)
Health economist	2 (5.3)	1 (3.6)
Veterinary scientists	2 (5.3)	1 (3.6)
Principal food safety scientist	1 (2.6)	1 (3.6)
**Jurisdiction of work** (***n***, %)		
Victoria	10 (26.3)	6 (21.4)
Queensland	8 (20.5)	7 (25.0)
Australian capital territory	7 (17.9)	6 (21.4)
New South Wales	6 (15.4)	4 (14.3)
Tasmania	4 (10.3)	3 (10.7)
South Australia	2 (5.3)	2 (7.4)
Northern Territory	1 (2.6)	–
**Highest education** (***n***, %)		
PhD	25 (63.2)	20 (71.4)
Master's degree	11 (28.9)	6 (21.4)
MBBS	2 (5.3)	1 (3.6)
Fellowship in pathology	1 (2.6)	1 (3.6)
**Experience in infectious disease control**. (years) (***n***, %)		
None	4 (10.5)	2 (7.1)
1–5 years	3 (7.9)	3 (10.7)
5–10 years	3 (7.9)	2 (7.1)
10–20 years	14 (36.8)	9 (32.1)
>20 years	14 (36.8)	12 (42.9)
**Experience in public health**. (years) (***n***, %)		
None	2 (5.3)	2 (7.1)
1–5 years	3 (7.9)	2 (7.1)
5–10 years	12 (31.6)	10 (35.7)
10–20 years	13 (34.2)	7 (25.0)
>20 years	8 (21.1)	7 (25.0)
**Experience in pathogen genomics**. (years) (***n***, %)		
None	4 (10.5)	3 (10.7)
<1 year	4 (10.5)	2 (7.1)
1–5 years	8 (21.1)	5 (17.9)
5–10 years	18 (47.4)	15 (53.6)
10–20 years	2 (5.3)	2 (7.1)
>20 years	2 (5.3)	3.6 (1)

Abbreviation: MBBS, Bachelor of Medicine and Bachelor of Surgery.

### Delphi survey— consensus statement analysis

Experts achieved consensus on 53 of 138 statements (38.4%) across both rounds (Fig. 1), comprising 25 unique statements from Round 1 and 28 from Round 2 (including both previously partial consensus statements that reached full consensus and new statements) (Supplementary Material). In this paper, we only discuss these consensus statements. The statements that achieved partial consensus or no consensus from both rounds are provided in the Supplementary Material.

Experts reached consensus on critical mechanisms for determining pathogen prioritisation for genomics (Table 2). There was strong agreement that pathogen prioritisation should sometimes occur at the national level, particularly for international threats, multi-jurisdictional outbreaks and nationally notifiable diseases. At the jurisdiction level, the decision to prioritise should be through a collaborative process involving public health laboratories, public health units, and jurisdictional governments. Experts endorsed biennial reviews of priority pathogens with flexibility for situation-based updates. Strong consensus was achieved for prioritising novel or emerging pathogens and those causing outbreaks at various scales when sequencing would benefit public health response. Experts also agreed that public health laboratories should conduct sequencing on biological samples from animal, agriculture and environmental sources, not just humans.

**Table 2:** Consensus on mechanisms and processes for determining priority pathogens for genomic surveillance.

Statements (number of responses)	Agreement index
**The following statements are about who should decide which pathogens should be prioritised for genome sequencing.**	
In some circumstances, the decision on which pathogens to prioritise for sequencing should be made at the national level (***n*** = 38)	0.92
Public health laboratories and public health units in each jurisdiction should jointly decide on which pathogens to prioritise for sequencing (***n*** = 28).	0.89
A nationally agreed list should be established for sequencing pathogens for routine surveillance, in consultation with state/territory governments and public health units (***n*** = 28).	0.89
**The following statements are about how often the pathogens that are sequenced should be reviewed and revised.**	
Pathogens for sequencing should be reviewed every two years with an option of periodic reviews in response to outbreaks, national priorities, and evolving situations (***n*** = 27).	0.89
Pathogens for sequencing to be reviewed as needed, based on the situation (***n*** = 37).	0.81
**The following statements are about public health laboratory's decisions to conduct pathogen sequencing.**	
Public Health Laboratories should conduct sequencing on all types of biological samples, including those obtained from animals, agriculture and the environment, in addition to human samples (***n*** = 27).	0.85∗
**The following statements are about the circumstances to decide which pathogens to prioritise for sequencing.**	
Novel or emerging pathogens should be prioritised for sequencing if the information will support the public health response (***n*** = 27).	0.93∗∗
Pathogens causing local, national, or international outbreaks should be prioritised for sequencing if the information will support the public health response (***n*** = 27).	0.93∗∗
Pathogens should be prioritised for sequencing when there is an outbreak in multiple countries or a notification from global health organisations such as the World Health Organization (WHO) or the US Centers for Disease Control and Prevention (US-CDC) (***n*** = 27).	0.89∗
Pathogens causing multijurisdictional outbreaks should be prioritised for sequencing (***n*** = 36).	0.86
Pathogens causing local outbreaks should be prioritised for sequencing (***n*** = 27).	0.82∗

Note: The Agreement Index represents the proportion of experts who agreed or disagreed with the statement. Numbers in parentheses show total respondents for each statement. Variation in respondent numbers reflects statements introduced/revised in Round 2 and exclusion of ‘no opinion’ responses from analysis. Cut-off for consensus was agreement index of ≥0.80 among experts agreeing or disagreeing with a statement. Agreement Index is 1. Rating on 5-point Likert scale with 1 as strongly agree and 5 as strongly disagree. Statements with ∗ shows rerating of original statement from round 1 which achieved consensus in round 2, statements with ∗∗ shows rating of revised/modified statement from a question round 1, which achieved consensus in round 2.

Experts reached consensus on various criteria for prioritising pathogens for genome sequencing based on both pathogen characteristics and disease epidemiology (Table 3). Among pathogen-related factors, there was particularly strong consensus for prioritising vector-borne pathogens with high public health impact, foodborne pathogens with potential for multi-jurisdictional spread, and airborne pathogens of high public health impact. Other important pathogen-related criteria included antimicrobial resistance, novel and emerging pathogens, and highly virulent pathogens.

**Table 3:** Consensus criteria for prioritising pathogens for genome sequencing: pathogen characteristics and disease epidemiology.

Statements (number of responses)	Agreement index
**Pathogen-related factors**	
Pathogens with a vector borne mode of transmission of high public health impact (***n*** = 24).	0.97∗∗
Pathogens with a foodborne mode of transmission with a potential for multi-jurisdictional spread (***n*** = 26).	0.96∗∗
Pathogens with an airborne mode of transmission of high public health impact (***n*** = 25).	0.96∗∗
Pathogens with a sexual mode of transmission if associated with a serious disease (***n*** = 25).	0.92∗∗
Pathogens associated with antimicrobial resistance (***n*** = 35).	0.91
Novel and emerging pathogens (***n*** = 34).	0.91
Pathogens that are highly virulent (***n*** = 33).	0.91
Pathogens with high human-to-human transmissibility (***n*** = 32).	0.84
Pathogens that are potential agents of bioterrorism (viruses, bacteria, toxins or other harmful agents to cause illness or death in people, animals or plants) (***n*** = 26).	0.89∗
Pathogens with a water-borne mode of transmission with a high public health impact (***n*** = 26).	0.89∗∗
Pathogens with the potential of causing spillover and spillback transmission (***n*** = 32).	0.88
Pathogens with no effective or widely available medical counter measure or treatment if the pathogen is highly transmissible (***n*** = 26).	0.88∗∗
Pathogens with a vector borne mode of transmission if highly pathogenic (***n*** = 25)∗∗	0.88∗∗
Pathogens with transmissibility during the incubation period if associated with serious disease (***n*** = 24).	0.83∗∗
Pathogens that are vaccine preventable (***n*** = 26).	0.81∗
**Epidemiological factors**	
Pathogens with the potential of causing significant infections in the institutional and hospital settings (***n*** = 31).	0.94
Pathogens with the potential to cause high morbidity (***n*** = 32).	0.91
Pathogens with the potential to cause many people to be hospitalised (***n*** = 32).	0.91
Pathogens disproportionately affecting Aboriginal and Torres Strait Islander populations (***n*** = 32).	0.91
Pathogens with the potential to cause high case fatality ratio (***n*** = 32).	0.88
Pathogens with the potential to adversely affect economic and trade activities at local, jurisdictional and/or national levels (***n*** = 24).	0.83∗
Pathogens that are notifiable (***n*** = 32).	0.81

Note: The Agreement Index represents the proportion of experts who prioritised the pathogen specific pathogen criteria. Variation in respondent numbers reflects statements introduced/revised in Round 2 and exclusion of ‘no opinion’ responses from analysis. Cut-off for consensus was agreement index of ≥0.80 among experts rating the statement as important or not important. Rating on 5-point Likert scale with 1 as extremely important and 5 as not important at all. Statements with ∗ shows rerating of original statement from round 1 which achieved consensus in round 2, statements with ∗∗ shows rating of revised/modified statement from a question round 1, which achieved consensus in round 2.

For epidemiological factors, experts strongly agreed on prioritising pathogens with potential to cause significant infections in institutional and hospital settings. There was also strong consensus on prioritising pathogens with potential to cause high morbidity, high hospitalisation rates, and those disproportionately affecting Aboriginal and Torres Strait Islander populations. Overall, 22 statements reached consensus across both pathogen-related and epidemiological categories, with agreement indices ranging from 0.81 to 0.97.

Table 4 presents expert consensus on pathogen prioritisation for genomic surveillance across three different surveillance contexts. For outbreak investigation, experts prioritised 11 pathogens including Carbapenemase-producing *Enterobacterales*, specific pathogenic *E. coli* subtypes (STEC, ETEC, EIEC, EAEC) and *Salmonella*. For routine surveillance, *Mycobacterium tuberculosis* received the highest consensus, followed by multidrug-resistant *Staphylococcus aureus*. Three pathogens, *Shigella*, *Neisseria gonorrhoeae* and invasive Group A *Streptococcus*, were prioritised for periodic analysis.

**Table 4:** Consensus on prioritising pathogens for genome sequencing in outbreak investigations and surveillance activities.

Definitions: Outbreak investigation: Identifying the source of ongoing outbreaks and preventing additional cases; Routine surveillance: Regular, ongoing systematic collection, analysis, and interpretation to monitor circulating pathogens and detect changes or trends over time. Periodic Surveillance: One-time analysis of representative samples at a specific point in time to determine what pathogens are currently circulating in a population.

Note: The Agreement Index represents the proportion of experts who prioritised the pathogen for the specific surveillance context. Variation in respondent numbers reflects statements introduced/revised in Round 2 and exclusion of ‘no opinion’ responses from analysis. Cut-off for consensus was agreement index of ≥0.80 among experts rating the genome sequencing as high priority or low priority for a given situation. Rating on 5-point Likert scale with 1 as highest priority and 5 as least priority. Values in parentheses show number of experts who agreed for each pathogen-context combination. Statements with ∗ shows rerating of original statement from round 1 which achieved consensus in round 2, statements with ∗∗ shows rating of revised/modified statement from a question round 1, which achieved consensus in round 2.

### Expert perspectives on pathogen prioritisation—thematic analysis of free-text responses

Our thematic analysis revealed two primary themes: Strategic Governance and Operational Implementation.

### Strategic governance

#### Balancing national and local priorities

Experts recognised the complexity of prioritising pathogens for sequencing in Australia where each jurisdiction may have different priorities. They emphasised the need for national level coordination of prioritisation which should accommodate jurisdiction-specific needs and resources.

*“I strongly agree there should be priority pathogens at a national level but would expect this to be a subset of pathogens that undergo genomic surveillance, with local needs dictating the others.”*

They also stressed the need for multi-stakeholder collaboration for decisions regarding prioritising for genomic surveillance across jurisdictions and at the national level including public health laboratories, public health units, infectious disease and prevention units, and end users like public health practitioners and national surveillance networks to prevent fragmented surveillance.

“*There should be shared decision making between labs and state departments of health with some influence from the Commonwealth and relevant national committees such as OzFoodNet.”*

Experts suggested that this integrated approach should consider varying priorities across stakeholders. Experts recommended regular dialogue between laboratories and health departments to align pathogen prioritisation with public health utility, response capacity, technical feasibility, and complementarity to existing methods.

*“…there will be priorities for the public health labs (e.g. pathogens with complex laboratory diagnostics where whole genome sequencing will provide significant process improvements, or where genomics has a role in clinical care, assisting diagnostics or infection control), there will be priorities for the public health units (e.g. pathogens where the epidemiology can be unclear, and whole genome sequencing will play a big role in informing public health decisions), and there will be joint priorities. I don't think there needs to be a single point of decision around prioritisation.”*

#### One health integration

Experts advocated for an integrated One Health approach, recommending public health laboratories as central sequencing points while emphasising the need for multidisciplinary expertise to properly interpret findings across human, animal, and environmental contexts.

“…*if there is no suitable environmental/agricultural department/lab to do the work then public health labs can fill this need—however it's critical that non-human focused viewpoints are considered— too often it seems that public health labs have a human only focus, and my fear would be that if they sequenced all samples then all samples would be viewed from this prism. It's not just about having a machine and a pipeline, but the expertise to unify the human/animal/environmental implications of findings.”*

### Practical implementation

#### Resource and system optimisation

Resource constraints heavily influenced prioritisation recommendations, with experts favouring actionable results while warning against system overload:

*“Ideally if it were possible to do all these different organisms that would be great as they have varying public health and clinical importance. Resourcing is crucial though…”*

Laboratory capabilities and resources were a recurrent theme in responses. Experts emphasised that while larger laboratories may have adequate resources and trained staff, such as bioinformaticians and technicians specialised in genome sequencing, these capabilities vary substantially between jurisdictions. When resources are limited, experts recommended prioritising based on result actionability rather than collecting “nice to know” information.

System overload emerged as a significant concern. One expert cautioned:

*“We need to be careful not to overload the system with high-volume whole genome sequencing for multiple pathogens, which could become a mass data collection exercise without leading to action.”*

Experts emphasised the importance of deliberate selection of pathogens that considers not just which pathogens to sequence but also the context of sequencing. One expert suggested

“*In many circumstances, it might be more beneficial to prioritise a broader range of pathogens, but fewer of each individual pathogen, especially when outbreak detection isn't the primary surveillance objective*.”

Together, these insights underscore the need for strategic sequencing frameworks that balance comprehensive surveillance with laboratory capacity while ensuring genomic data translates to meaningful public health action.

#### Public health impact and feasibility

The multifaceted nature of prioritisation decision-making was widely acknowledged by the experts. Experts advocated for evaluation frameworks that integrate multiple factors: potential for actionable public health interventions, overall disease burden, data quality and availability, and specific information needs for policy development. As one expert articulated

“*For most, whether public health genomics is a priority depends on the feasibility of responses or interventions, given prevalence, availability of epidemiological data, and elements requiring monitoring to inform policy*.”

## Discussion

This expert elicitation, drawing on insights from experienced infectious disease specialists across Australia, highlights mechanisms, processes, general criteria and specific circumstances for prioritising pathogens for genome sequencing in Australia. Our integrated approach ensured both systematic framework development and practical validation of prioritisation decision-making. While our study focused on bacterial pathogens, the consensus criteria we established—such as novel and emerging pathogens, antimicrobial resistance, and high public health impact—are applicable across all pathogen types including viruses, fungi, and parasites, and support strain-specific prioritisation based on resistance patterns or serotypes. The governance approach identified by our experts aligns with Strategic Priority 1 of the Australian National Microbial Genomics Framework 2025–2027.^21^ Our experts recommended multidisciplinary collaborative prioritisation at the national level for international threats, multi-jurisdictional outbreaks, and nationally notifiable diseases, while preserving jurisdictional autonomy and implementing biennial reviews with situation-based flexibility. The consensus statements will form a systematic framework for pathogen prioritisation in Australia, enabling consistent decision-making and evidence-based resource allocation across jurisdictions. These results will guide development of a comprehensive national prioritisation framework. This approach supports a coordinated and consistent system-focused application of microbial genomics in public health practice across all states and territories.

Most of epidemiological and disease specific characteristics identified by our experts have been reported in global literature.^9^^,^^10^^,^^13^^,^^26^ While German experts prioritised case fatality rate,^10^ Australian experts gave higher rating to prioritising pathogens causing institutional and hospital infections, those resulting in high morbidity and hospitalisations, and pathogens disproportionately affecting Aboriginal and Torres Strait Islander populations. This reflects Australia's unique epidemiological context, and a focus on health equity. Similarly, highest consensus emerged for vector-borne and airborne pathogens with high public health impact, reflecting growing concerns of Australian experts about emerging climate-sensitive disease threats.^27^^,^^28^ These priorities also align with the National Microbial Genomics Framework 2025–2027 One Health approach to address environmental, animal and human health threats and to identify priority organisms of national significance.^21^

Our experts' recommendations for surveillance-specific prioritisation of pathogens aligned with international literature. Prioritisation of *Listeria monocytogenes, Mycobacterium tuberculosis, Neisseria meningitidis,* and *Salmonella* for both outbreak investigations and routine surveillance parallels the ECDC's strategic framework.^26^ Tuberculosis received strong consensus across multiple studies for prioritising for outbreak and routine surveillance,^10^^,^^12^^,^^13^ reflecting global concern about its public health threat to human health. Despite Australia's low Tuberculosis incidence, vulnerable populations including overseas-born persons from high-incidence countries, Aboriginal and Torres Strait Islander peoples and immunocompromised individuals remain at risk,^29^ likely explaining our experts' prioritisation for routine surveillance. Greater emphasis on foodborne pathogens reflects Australia's significant burden from foodborne illness,^30^ demonstrating how local epidemiology influences surveillance priorities.

Our qualitative analysis identified strategic governance and practical implementation as central themes contextualising the quantitative findings. Perspectives on balancing priorities revealed the importance of collaborative processes for genomic surveillance that respect jurisdictional autonomy while ensuring nationally consistent approaches for critical pathogens in special circumstances. The One Health theme highlighted that while public health laboratories may serve as central sequencing points, multidisciplinary expertise remains essential. Both of these expert's perspectives reflect what has been suggested in the Australian National Microbial Genomics Framework 2025–2027.^21^

Within the practical implementation theme, resource constraints emerged as a particularly significant consideration. In agreement with previous evidence, experts emphasised that limited resources remain a critical barrier to routine pathogen genomics implementation.^4^^,^^18^^,^^21^ This targeted approach provides an effective interim strategy until sustainable funding models emerge or technological advances drive sequencing costs down significantly.

An effective genomic-based public health response requires active collaboration among diverse stakeholders including public health laboratories, health departments, academia, animal health, agriculture and environmental health departments, public health practitioners and the private sector locally and at international levels.^4^ This multi-stakeholder approach is particularly relevant in Australia's federated health system, where states and territories independently manage public health activities.^19^ The recently established Australian Centre for Disease Control (ACDC) could serve as an ideal coordinating body for implementing the governance framework identified by our experts while supporting transmission tracking, outbreak investigation and antimicrobial stewardship. With its mandate to respond to infectious disease threats, the ACDC is well-positioned to facilitate successful implementation of collaborative pathogen prioritisation at national level while respecting jurisdictional autonomy,^31^ creating surveillance systems responsive to Australia's unique ecological and epidemiological landscape.

### Strengths and limitations

We utilised a rigorous mixed-methods approach in this study, combining quantitative consensus with qualitative analysis, enabling comprehensive understanding of expert perspectives. This methodological triangulation strengthened validity and captured implementation complexity. Our diverse expert panel ensured priorities balanced technical feasibility with public health relevance. Our rating-based approach using an Agreement Index provided transparency by showing the exact proportions of expert agreement (e.g., 0.97 vs 0.81), unlike a simple ranking which only shows relative order. This revealed consensus strength and identified areas where experts disagreed, providing a more nuanced understanding of prioritisation perspectives.

Limitations of our study include a prolonged data collection period and lack of representation from one Australian jurisdiction, meaning some jurisdiction-specific considerations may not be fully reflected. However, as we had representation from all other Australian jurisdictions, we consider that the opinions expressed by the experts reflected views from all of Australia. The use of subjective terms such as ‘high public health impact’ and ‘would benefit public health response’ may have introduced interpretive variability among participants. While these terms emerged from expert consensus in Round 1 and achieved high agreement in subsequent rounds, different experts may have weighted factors such as disease severity, infectiousness, healthcare capacity, and resource requirements differently when making their assessments. Additionally, some consensus statements exhibit overlapping characteristics (e.g., virulence, hospitalisation potential, case fatality ratios), which enable nuanced pathogen categorisation rather than redundancy, as different pathogens may score variably across these related but distinct criteria.

### Future directions

This expert elicitation provides a foundation for implementing coordinated pathogen genomic surveillance in Australia with potential application to other federated health systems. The results of this expert elicitation will be used to develop a framework for pathogen prioritisation in Australia. Our approach may prove a useful model for other countries considering how to prioritise pathogens for whole genome sequencing as part of improvements to public health surveillance.

## Contributors

Contributors TZ: Conceptualization, methodology, data curation, investigation, formal analysis and writing (original draft and reviewing), verification of the data, had access to raw data, final responsibility for submission.

AF: Conceptualization, methodology, investigation, data curation, and writing (review & editing), data verification.

DC, NM: data curation, data verification, had access to raw data, methodology, investigation, and writing (review & editing).

SN: Writing (review & editing).

PA and AZ: Data curation, investigation, and writing (review & editing).

ST: project administration, and writing (review & editing).

KG, MDK: Funding acquisition, Conceptualization, methodology, supervision, and writing (review & editing).

BPH: Funding acquisition, and writing (review & editing).

## Data sharing statement

Deidentified data are available by request to the corresponding author.

## Ethics statement

The ANU Science and Medical Delegated Ethical Review Committee approved this study (protocol 2022/407).

## AI statement

Generative AI was used to assist in creating the Delphi process flowchart (Fig. 1). Claude 3.7 Sonnet (March 2025) was used to generate an initial Mermaid diagram based on the study data. The prompt used was: ‘Create a Mermaid flowchart showing a two-round Delphi survey process with the following data: Round 1 had 87 statements total with 28.7% (n = 25) achieving full consensus (AI >0.80), 32.2% (n = 28) achieving partial consensus (AI 0.60–0.79), and 41.8% (n = 36) having no consensus (AI <0.59). Statements with partial consensus proceeded to Round 2, which had 47 statements with 63.8% (n = 30) achieving full consensus, 8.5% (n = 4) achieving partial consensus, and 27.6% (n = 13) having no consensus.’ The generated code was exported to the Mermaid Live Editor (https://mermaid.live/) where it was manually edited to enhance layout, styling, and text clarity. The output was reviewed and edited by the corresponding author (TZ) to ensure accuracy in representing the methodology employed in this study. The authors take full responsibility for the content and accuracy of the figure.

## Declaration of interests

This research was supported by the Australian National Health and Medical Research Council, Medical Research Futures Fund (FSPGN00049) as part of the AusPathoGen program. BPH received an Investigator Grant (GNT1196103) from the Australian National Health and Medical Research Council. The funders had no role in study design, data collection and analysis, decision to publish, or preparation of the manuscript. The authors declare no other competing interests.
