# Supplementary material for: Insights from expert elicitation: prioritising pathogens for genome sequencing for Australian public health response
Source: Lancet Reg Health West Pac. 2025 Aug 25;62:101670. doi: 10.1016/j.lanwpc.2025.101670 (PMC12541144; doi:10.1016/j.lanwpc.2025.101670)
Supplement: Supplementary Data [file mmc1.docx]

**Supplementary Tables: Delphi Survey Results**

**List of Priority Pathogens Evaluated in Expert Elicitation**

**Round 1**

1. *Shigella* spp.
2. *Neisseria gonorrhoeae*
3. *Salmonella* spp.
4. *Listeria monocytogenes*
5. *Mycobacterium tuberculosis*
6. Group A *streptococcus*
7. *Staphylococcus aureus*
8. *Burkholderia pseudomallei*
9. *Escherichia coli*
10. *Escherichia coli* (STEC, ETEC, EIEC, EAEC)
11. Carbapenamase-producing enterobacterales
12. *Neisseria meningitidis*
13. *Streptococcus pneumoniae*
14. Measles
15. Influenza

**Round 2 Addition (total n=16)**

*Added based on expert suggestions from Round 1 free-text responses*

1. Invasive Group A *Streptococcus* (iGAS)

**Notes**

1. The original 14 AusPathoGen priority pathogens were predetermined at the inception of the AusPathoGen project based on high disease burden, frequency as cause of outbreaks, difficulty in investigation and control, spread to high-risk and vulnerable populations, or resistance to almost all available antimicrobials.
2. *Escherichia coli* was included both as a broad category (entry 9) and as specific pathogenic subtypes (entry 10) to allow experts to differentiate between general surveillance needs and pathotype-specific priorities**.**
3. Invasive Group A *Streptococcus* was added in Round 2 following expert recommendations to distinguish invasive from non-invasive Group A *Streptococcus* infection for genomic surveillance prioritisation**.**

**Table S1. Statements on Mechanisms and Processes for Determining Priority Pathogens for Whole Genome Sequencing from Round 1 that Achieved Consensus in Round 2**

| Statements | Round 1 Agreement Index (n) | Round 2 Agreement Index (n) | Consensus Type | Change |
| --- | --- | --- | --- | --- |
| Decision-making authority for pathogen prioritisation |  |  |  |  |
| Public health laboratories and public health units in each jurisdiction should jointly decide on which pathogens to prioritise for WGS* | 0.61 (37) | 0.89 (28) | Agreement | +0.28 |
| A nationally agreed list should be established for sequencing pathogens for routine surveillance, in consultation with state/territory governments and public health units† | — | 0.89 (28) | Agreement | New |
| Review frequency for sequencing priorities |  |  |  |  |
| Pathogens for sequencing should be reviewed every two years with an option of periodic reviews in response to outbreaks, national priorities, and evolving situations† | — | 0.89 (27) | Agreement | New |
| Laboratory scope for WGS implementation |  |  |  |  |
| *Public health laboratories should conduct WGS exclusively on samples obtained from humans** | 0.76 (37) | 0.85 (27) | Disagreement | +0.09 |
| Public health laboratories should conduct WGS on all types of biological samples, including those obtained from animals, agriculture, and the environment, in addition to human samples* | 0.66 (35) | 0.85 (27) | Agreement | +0.19 |
| Circumstances for pathogen prioritisation |  |  |  |  |
| Pathogens should be prioritised for WGS when there is an outbreak in multiple countries or a notification from global health organisations such as WHO or US-CDC** | 0.78 (37) | 0.89 (27) | Agreement | +0.11 |
| Pathogens causing local outbreaks should be prioritised for WGS* | 0.63 (35) | 0.82 (27) | Agreement | +0.19 |
| New statements introduced in Round 2 which achieved consensus |  |  |  |  |
| Novel or emerging pathogens should be prioritised for sequencing if the information will support the public health response† | — | 0.93 (27) | Agreement | New |
| Pathogens causing local, national, or international outbreaks should be prioritised for sequencing if the information will support the public health response† | — | 0.93 (27) | Agreement | New |

**Note:** The Agreement Index represents the proportion of experts who agreed or disagreed with the statement. Numbers in parentheses show total respondents for each statement. Variation in respondent numbers reflects statements introduced/revised in Round 2 and exclusion of 'no opinion' responses from analysis. Cut-off for consensus was agreement index of > 0.80 among experts agreeing or disagreeing with a statement. Agreement Index ranges from 0 to 1. Rating on 5-point Likert scale with 1 as strongly agree and 5 as strongly disagree.

*Statement re-rated from Round 1 that achieved consensus in Round 2
** Revised/modified statement from Round 1 that achieved consensus in Round 2
† New statement introduced in Round 2
Statements in italics are those where consensus was on disagreement

WGS = Whole Genome Sequencing; WHO = World Health Organization; US-CDC = United States Centers for Disease Control and Prevention

**Table S2. Pathogen-Specific and Epidemiological Factors for Whole Genome Sequencing from Round 1 that Achieved Consensus in Round 2**

| Statements | Round 1 Agreement Index (n) | Round 2 Agreement Index (n) | Consensus Type | Change |
| --- | --- | --- | --- | --- |
| Pathogen-related factors |  |  |  |  |
| Vector borne pathogens of high public health impact† | — | 0.97 (24) | Agreement | New |
| Pathogens with a foodborne mode of transmission with potential for multi-jurisdictional spread‡ | — | 0.96 (26) | Agreement | Extended |
| Pathogens with an airborne mode of transmission of high public health impact‡ | — | 0.96 (25) | Agreement | Extended |
| Sexual transmission pathogens if associated with serious disease‡ | — | 0.92 (25) | Agreement | Extended |
| Pathogens that are potential agents of bioterrorism* | 0.79 (34) | 0.89 (26) | Agreement | +0.10 |
| Pathogens with water-borne mode of transmission with high public health impact† | — | 0.89 (26) | Agreement | New |
| Vector borne pathogens if highly pathogenic‡ | — | 0.88 (25) | Agreement | Extended |
| Pathogens that are vaccine preventable* | 0.74 (31) | 0.81 (26) | Agreement | +0.07 |
| Pathogens with a foodborne mode of transmission | 0.79 (33) | — | — | — |
| Pathogens with a vector borne mode of transmission | 0.70 (33) | — | — | — |
| Pathogens with a sexual mode of transmission | 0.72 (32) | — | — | — |
| Pathogens with transmissibility during the incubation period | 0.65 (31) | — | — | — |
| Pathogens with transmissibility during incubation if associated with serious disease‡ | — | 0.83 (24) | Agreement | Extended |
| Pathogens with no effective or widely available medical countermeasure or treatment | 0.67 (27) | — | — | — |
| Pathogens with no effective treatment if highly transmissible‡ | — | 0.88 (26) | Agreement | Extended |
| Epidemiological factors |  |  |  |  |
| Pathogens with potential to adversely affect economic and trade activities* | 0.77 (30) | 0.83 (24) | Agreement | +0.06 |

**Note:** The Agreement Index represents the proportion of experts who agreed or disagreed with the statement. Numbers in parentheses show total respondents for each statement. Variation in respondent numbers reflects statements introduced/revised in Round 2 and exclusion of 'no opinion' responses from analysis. Cut-off for consensus was agreement index of > 0.80 among experts agreeing or disagreeing with a statement. Agreement Index ranges from 0 to 1. Rating on 5-point Likert scale with 1 as very important and 5 as very not important.

*Statement re-rated from Round 1 that achieved consensus in Round 2
† New statement introduced in Round 2
‡ Statement extended/refined from Round 1 with additional qualifying criteria

WGS = Whole Genome Sequencing

**Table S3. Pathogen Prioritisation for Whole Genome Sequencing during Outbreak, Routine, and Snapshot Surveillance from Round 1 that Achieved Consensus in Round 2**

| Pathogen | Surveillance Type | Round 1 Agreement Index (n) | Round 2 Agreement Index (n) | Consensus Type | Change |
| --- | --- | --- | --- | --- | --- |
| N. Gonorrhoea | Outbreak investigation* | 0.77 (26) | 0.80 (20) | Agreement | +0.03 |
| Measles | Outbreak investigation* | 0.70 (30) | 0.83 (23) | Agreement | +0.13 |
| Invasive Group A streptococcus (IGAS) | Outbreak investigation† | — | 0.91 (21) | Agreement | New |
| Salmonella | Routine surveillance* | 0.65 (31) | 0.83 (24) | Agreement | +0.18 |
| Listeria monocytogenes | Routine surveillance* | 0.68 (28) | 0.83 (24) | Agreement | +0.15 |
| Mycobacterium tuberculosis | Routine surveillance* | 0.75 (28) | 0.91 (23) | Agreement | +0.16 |
| Neisseria meningitidis | Routine surveillance* | 0.62 (26) | 0.82 (22) | Agreement | +0.20 |
| Invasive Group A streptococcus (IGAS) | Snapshot analysis† | — | 0.85 (20) | Agreement | New |
| Shigella | Snapshot analysis* | 0.67 (30) | 0.83 (23) | Agreement | +0.16 |

**Note:** The Agreement Index represents the proportion of experts who agreed or disagreed with the statement. Numbers in parentheses show total respondents for each statement. Variation in respondent numbers reflects statements introduced/revised in Round 2 and exclusion of 'no opinion' responses from analysis. Cut-off for consensus was agreement index of > 0.80 among experts agreeing or disagreeing with a statement. Agreement Index ranges from 0 to 1. Rating on 5-point Likert scale with 1 as high priority and 5 as low priority.

*Statement re-rated from Round 1 that achieved consensus in Round 2
† New pathogens introduced in Round 2

WGS = Whole Genome Sequencing; IGAS = Invasive Group A Streptococcus

**Table S4. Statements on Mechanisms and Processes for Determining Priority Pathogens for Whole Genome Sequencing (WGS) that Did Not Achieve Consensus in Round 1 and 2**

| Statements | Round 1 Agreement Index (n) | Round 2 Agreement Index (n) | Consensus Type | Change |
| --- | --- | --- | --- | --- |
| Decision-making authority for pathogen prioritisation |  |  |  |  |
| Determining priority pathogens for WGS should be the responsibility of national committees for centralised decision-making | 0.61 (26) | 0.62 (26) | Partial Agreement | +0.01 |
| Whole genome sequencing should be routinely conducted in all public health laboratories without the need for prioritisation | 0.64 (28) | 0.64 (28) | Partial Disagreement | 0.00 |
| Laboratory scope for WGS implementation |  |  |  |  |
| All public health laboratories should have the capability to sequence pathogens from human and non-human samples | 0.74 (20) | 0.74 (27) | Partial Agreement | 0.00 |
| Circumstances for pathogen prioritisation |  |  |  |  |
| All novel or emerging pathogens should be prioritised for WGS* | 0.78 (36) | 0.78 (27) | Partial Agreement | 0.00 |

**Note:** The Agreement Index represents the proportion of experts who agreed or disagreed with the statement. Numbers in parentheses show total respondents for each statement. Cut-off for partial consensus was agreement index of 0.60-0.79 among experts agreeing or disagreeing with a statement. Full consensus required agreement index of > 0.80. Agreement Index ranges from 0 to 1. Rating on 5-point Likert scale with 1 as strongly agree and 5 as strongly disagree. Statements in italics are those where consensus was on disagreement.

*Statement re-rated from Round 1

WGS = Whole Genome Sequencing

**Table S5. Pathogen-Specific and Epidemiological Factors for Whole Genome Sequencing that Did Not Achieve Consensus in Round 1 and 2**

| Factor | Round 1 Agreement Index (n) | Round 2 Agreement Index (n) | Consensus Type | Change |
| --- | --- | --- | --- | --- |
| Pathogen-related factors |  |  |  |  |
| Pathogens that are not vaccine preventable* | 0.63 (30) | 0.76 (25) | Partial Agreement | +0.13 |
| Pathogens with a foodborne mode of transmission* | 0.79 (33) | 0.79 (24) | Partial Agreement | 0.00 |
| Pathogens with an airborne mode of transmission* | 0.61 (31) | 0.62 (26) | Partial Agreement | +0.01 |
| Pathogens with a vector borne mode of transmission* | 0.70 (33) | 0.72 (25) | Partial Agreement | +0.03 |
| Vector borne pathogens associated with climate change‡ | — | 0.65 (23) | Partial Agreement | Extended |
| Pathogens with a sexual mode of transmission* | 0.72 (32) | 0.74 (27) | Partial Agreement | +0.03 |
| Pathogens with transmissibility during the incubation period* | 0.65 (31) | 0.67 (24) | Partial Agreement | +0.02 |
| Pathogens with no effective or widely available medical countermeasure or treatment | 0.67 (27) | 0.68 (25) | Partial Agreement | +0.01 |
| Pathogens with no effective treatment if associated with serious disease‡ | — | 0.72 (25) | Partial Agreement | Extended |
| Pathogens with vertical transmission if on notifiable disease list† | — | 0.67 (24) | Partial Agreement | New |
| Pathogens with vertical transmission if associated with serious disease† | — | 0.79 (24) | Partial Agreement | New |
| Epidemiological factors |  |  |  |  |
| Pathogens disproportionately affecting children, pregnant women, and the elderly* | 0.63 (32) | 0.62 (26) | Partial Agreement | -0.01 |
| Pathogens with potential to cause high rates of hospitalisation in people with chronic conditions or other comorbidities† | — | 0.67 (24) | Partial Agreement | New |

**Note:** The Agreement Index represents the proportion of experts who agreed or disagreed with the statement. Numbers in parentheses show total respondents for each statement. Cut-off for partial consensus was agreement index of 0.60-0.79 among experts agreeing or disagreeing with a statement. Full consensus required agreement index of > 0.80. Agreement Index ranges from 0 to 1. Rating on 5-point Likert scale with 1 as very important and 5 as very not important.

*Statement re-rated from Round 1
† New statement introduced in Round 2
‡ Statement extended/refined from Round 1 with additional qualifying criteria

WGS = Whole Genome Sequencing

**Table S6. Pathogen Prioritisation for Whole Genome Sequencing during Outbreak, Routine, and Snapshot Surveillance that Did Not Achieve Consensus in Round 1 and 2**

| Pathogen | Surveillance Type | Round 1 Agreement Index (n) | Round 2 Agreement Index (n) | Consensus Type | Change |
| --- | --- | --- | --- | --- | --- |
| *Shigella* | *Snapshot analysis** | 0.67 (30) | 0.65 (20) | Partial Agreement | -0.02 |
| *Shigella* | *Snapshot analysis for high-risk groups (men who have sex with men)‡* | — | 0.64 (22) | Partial Agreement | Extended |
| *Carbapenamase-producing enterobacterales (CPE)* | *Snapshot analysis** | 0.68 (28) | 0.65 (20) | Partial Agreement | -0.03 |
| *Streptococcus pneumoniae* | *Outbreak investigation** | 0.70 (27) | 0.68 (22) | Partial Agreement | -0.02 |
| *Escherichia coli* | *Routine surveillance when associated with severe or invasive disease†* | — | 0.65 (23) | Partial Agreement | New |

**Note:** The Agreement Index represents the proportion of experts who agreed or disagreed with the statement. Numbers in parentheses show total respondents for each statement. Cut-off for partial consensus was agreement index of 0.60-0.79 among experts agreeing or disagreeing with a statement. Full consensus required agreement index of > 0.80. Agreement Index ranges from 0 to 1. Rating on 5-point Likert scale with 1 as high priority and 5 as low priority.

*Statement re-rated from Round 1
† New statement introduced in Round 2
‡ Statement extended/refined from Round 1 with additional qualifying criteria
Statements in italics represent partial consensus

WGS = Whole Genome Sequencing; CPE = Carbapenamase-producing Enterobacterales

**Code for the mermaid diagram**

[**Mermaid | Diagramming and charting tool**](https://mermaid.js.org/)

flowchart TD

title["Expert Elicitation

Delphi Survey Process"]

%% Round 1

A[Round 1

Statements included in Delphi survey = 89] --> B{Round 1}

B -->|"28.1% (n=25)"| C[Full Consensus Statements

AI≥ 0.80]

B -->|"31.5% (n=28)"| D[Partial Consensus Statements

AI 0.60-0.79

Included in round 2 for second opinion]

B -->|"40.4% (n=36)"| E[No consensus Statements

AI <0.59

Excluded from round 2]

%% Round 2

D --> F[Round 2

Statements included in\Delphi survey = 49]

F --> G{Round 2}

G -->|"61.2% (n=28)"| H[Full Consensus Statements

AI≥ 0.80]

G -->|"30.6% (n=15)"| I[Partial Consensus Statements

AI 0.60-0.79]

G -->|"8.2% (n=6)"| J[No consensus statements

AI <0.59 ]

%% Styling

classDef title font-size:20px,font-weight:bold

classDef round1 fill:#f0f8ff,stroke:#333

classDef round2 fill:#e6f3ff,stroke:#333

classDef consensus fill:#2a9d8f,color:white,stroke:#333

classDef partialConsensus fill:#457b9d,color:white,stroke:#333

classDef noConsensus fill:#e63946,color:white,stroke:#333

classDef evaluation fill:#a8dadc,color:#1d3557,stroke:#333

class title title

class A,B round1

class F,G round2

class C,H consensus

class D,I partialConsensus

class E,J noConsensus

class B,G evaluation
